# Modelling the Archipelago: Corfu as a Case Study for a Digital Edition of Cristoforo Buondelmonti’s *Liber Insularum*

**DOI:** 10.12688/openreseurope.16712.1

**Published:** 2024-01-08

**Authors:** Benedetta Bessi, Daniele Fusi

**Affiliations:** 1 Department of the Humanities, Ca' Foscari University of Venice, Venice, Veneto, 30123, Italy

**Keywords:** digital humanities; digital edition; spatial history; mapping; classical archaeology; history of archaeology; isolaria

## Abstract

The *Liber Insularum* by Cristoforo Buondelmonti can be considered the first guide to the Greek islands, each of them described by a textual paragraph and illustrated by color maps, in a format which gave rise to the new literary genre of *Isolaria*.” Mapping the Aegean: Cristoforo Buondelmonti’s *Liber Insularum”* is a Marie Skłodowska-Curie project aimed at the study of this book. This paper illustrates the application of Cadmus, a structured content management tool, to the creation of a digital edition of the *Liber* and to do this, we focus on the text and map of Corfu as a case study. After a historical introduction on the author and his work and the presentation of the project, we explain why we chose to use this tool and its main characteristics, and we offer a concrete example of its application to the material pertaining to the description of Corfu by showing its frontend output.

## Historical introduction

Cristoforo was born in Florence probably between 1380–1390 from a branch of the noble Buondelmonti family. His self-qualifications and references indicate that while in Florence he pursued an ecclesiastic career as presbyter of the church Santa Maria Sopr’Arno. During his early years, he was exposed to the teaching of prominent humanists such as Domenico Bandini and Coluccio Salutati, this latter directly involved in the promotion of Greek Studies in Florence. In 1397, Salutati, chancelor of the Florentine Republic, created the first public chair of Greek Studies in the West by appointing the Byzantine scholar, Manuel Chrysoloras, as teacher of Greek in the Studio, a move which contributed to the training of a new generation of humanists and to a general interest for ancient Greece and its heritage. Around 1414, Buondelmonti left Florence and moved to Rhodes, at that time seat of the Knights of St. John. The latest attestation for his presence on the island goes back to 1430, when he is mentioned in some archival documents as the dean of the Latin cathedral on the island. No information is known about his death and whether he ever went back to Florence (on the life of Buondelmonti: Barsanti, 2001; passim; Ragone, 2002, 184–193; Roger, 2012; Bessi, 2014, 223–228; Bessi, 2023, 64–67).

During his time in Greece, Buondelmonti travelled extensively through the Aegean exploring its islands and some places of Greece’s mainland and the coast of Asia Minor. The results of his travels were collected in two works: the *Descriptio Insule Crete* and the *Liber Insularum Archipelagi*.

The *Descriptio*, edited in multiple versions by Buondelmonti himself (1417, 1422/23, 1425/27), was dedicated to the Florentine humanist and manuscript collector Niccolò Niccoli and, as the name implies, it featured a detailed description of Crete based on the author’s visit and circumnavigation ( Bartelloni, 2021; Van Spitael, 1981).

The *Liber Insularum* was a *liber figuratus* (a picture book) with a format that combining textual descriptions of the islands and corresponding maps, is considered the forerunner of the new literary genre of the Isolarii. The Greek islands are described starting from the westernmost group of the Ionian islands and ending with Egina. Exception to this insularity are the descriptions of Gallipoli on the Strait of Hellespont, Constantinople and Mounth Athos. As shown in the acronym formed by the initial letters of each paragraph and by several references throughout the text, the *Liber* was dedicated to Cardinal Giordano Orsini, another protagonist of the Italian Humanism and an avid book collector with an interest in Greek Studies. The book circulated in various editions, a circumstance which has made the history of the tradition of the text quite complicated. As a matter of fact, scholars have reconstructed the existence of at least three, possibly four different editions of the *Liber* dating within a period comprehended between sometime before 1420 and 1430. Of these editions, the one dated to 1422 became the most popular and it is today referred to as the vulgata or standard text. Even considering the possibility that based on its shorter text, it could be an abridged version prepared by someone else rather than the author, it is undeniable that given its popularity, it is in this format that the Liber circulated in many copies through Italy and Europe influencing several generations of scholars and opening the path to new travels and explorations of Greece. More than 70 manuscript copies dating between the 15th and 16th century, are attested and even if its original version was in Latin, it was also quickly translated into Italian, English, French and Greek ( Pontari, 2013, 88–89; Ragone, 2002, 195–203).

At present no scientific critical edition of the *Liber* is available and the text is accessible only through the publication of single or arbitrarily collated manuscripts ( De Sinner, 1824; Legrand, 1897; Siebert & Plasman, 2005; Bayer, 2007; Edson, 2018).

Since the beginning of the 20th century, Buondelmonti has received the attention of scholars initially focused especially in the geographical and cartographic aspects of his books. In a second moment, the antiquarian value of his travels has also been recognized: Buondelmonti has been called an “ *umanista antiquario*” ( Weiss, 1964) and considered a pioneer in the rediscovery of Greek antiquities ( Beschi, 1986; Bessi, 2012). References to Buondelmonti’s witnesses are now a staple component in books and articles focusing on the history of the various Greek islands but no comprehensive commentary of his major work, the *Liber Insularum* has yet been published.

## Description of the project

“Mapping the Aegean: Cristoforo Buondelmonti’s *Liber Insularum* and the Birth of Classical Archaeology” is a three-year Marie Skłodowska-Curie project aimed at the study of the *Liber Insularum* and at the valorization of its role in the history of European cultural heritage since the travels of Buondelmonti paved the way to the rediscovery and exploration of Greece, a land whose territory, language and culture were still largely unknown to Western European scholars. An important component of the project is represented by the creation of a digital edition of the text based on a manuscript copy of the *Liber* kept in the Gennadios Library (MS Gennadios, Athens 71) and accompanied by an English translation and a detailed geographical, historical, and archaeological commentary (on the theories and practices of digital edition, Mancinelli-Pierazzo, 2020).

A digital edition here not only recommends itself to make this rare material be available to the widest possible audience; but also in consideration of its highly peculiar and multidisciplinary nature, which potentially requires the representation of a lot of heterogeneous and multimedial data, ranging from geography to history, from archaeology to literature, etc., and allowing interactive user experiences in accessing the figure maps and their various annotations. Further, the intrinsically open-ended nature of any digital edition lends itself to potentially relevant expansions, either from the contribution of single, specialized scholars, and from interested or local communities.

Even if, as we have seen above, a critical edition remains a desideratum in the scholarship on Buondelmonti, and digital methods and tools for working with manuscripts and witnesses, such as automatic text recognition, transcription, and collation, would make those tasks easier, the philological analysis of the text remains beyond the primary goal of MapAeg. The scope of this project is rather to highlight and to enhance the importance of the *Liber* as a document of the early travels in the Greek islands and its role in paving the way to the archaeological rediscovery of ancient Greece. Accordingly, the digital edition has been conceived as an easily accessible gateway to access the text and the related maps by the community of classical archaeologists but also scholars of classical studies, Byzantinists and historians of other periods who share an academic interest in the Greek islands. Striving at creating a user friendly and intuitive front-end experience, we also hope that the edition can be fruitfully used by a wider public made by instructors, students, independent travellers and stakeholders in the promotion of cultural tourism and community development.

For this reason, leaving behind the codicological and philological aspects of the research, we opted to produce our digital edition focusing on one single manuscript, the Gennadios, Athens, *ms.* 71. Although the choice can appear arbitrary, there are various reasons that motivated our decision. The Gennadius manuscript is a 15th century codex which contains the full textual description and a complete set of good quality maps with detailed annotations (not every manuscript of those attesting the *Liber* is in fact complete to this extent). Its text corresponds to the so called *vulgata*, which, as we have seen, even if at risk to be a non-authorial abridged version, was undeniably the most popular one and for sure the one through which the Liber was accessed by wide circles of scholars and humanists in Italy and Europe. The colour palette used in the maps of this manuscript is very close to that of the original which, as explained by Buondelmonti himself in the dedicatory introduction of his work to Giordano Orsini, was also very simple: “So that you can comprehend everything, in black mountains, in white plains, in green waters,”. Even though the Gennadios the mountains are rendered in brown and black is used for the outline of the islands and other geographical features (including mountains), the limited color range appears still close to the original trichromy especially when compared to other fancier and more elaborated specimens whose maps are coloured in more nuanced assortments which may include yellow, red, pink, blue, turquoise and even gold, or to those very simplified ones only black ink is used. Furthermore, the Gennadios specimen, given its location in one of the most important research libraries in Athens, is the manuscript through which, at least prior to the digital revolution, scholars and archaeologists interested in the Greek islands and gravitating around Athens and its research resources, have familiarized with Buondelmonti’s text. It is often maps from this manuscript which have been chosen to illustrate articles and books regarding the geography, the history, and the archaeology of the Greek islands. 

The Latin manuscript text has been transcribed in a semi-diplomatic format which maintains the original spelling, punctuation, and capitalization. Abbreviations are expanded. Textual notes accompany the English translation only when the original Latin text presents problems of intelligibility or considerable variations from the other edited texts. The English translation remains close to the Latin original but modern punctuation has been inserted. Length of sentences has been reduced and the syntax has been adapted to more modern usage even if we strive to maintain the richness and the complexity of the original vocabulary and phrasing as much as it was possible.

In line with the interests at the roots of this research and keeping in mind the final destinataries of the project (archaeologists, classicists, Byzantinists), particular attention has been given to the identification and discussion of the following elements:

- Etymologies of the island names and, when present, the mythological narrative that explains them.
- Geography, mainly orientation issues and presence of physical features such as, for example, mountains, springs, rivers, lakes, marshlands, gulfs, islets *etc*.
- Ancient monuments and ruins.
- Pagan cults and mythological episodes.
- Historical characters and episodes.
- Settlements and architectural structures such as towns, villages, castles, towers.
- Christian monuments such as monasteries and churches.
- Local flora and fauna.
- Trade and economy.
- Local lifestyle and folklore.

For all the above, a careful analysis of the text has been carried out to identify any ancient or medieval source used by Buondelmonti, either in the form of direct quotes or loose paraphrases of their content.

Since this research stems out of an archaeological approach to the *Liber*, the terms “geographical” and “geography” (as well as the somehow related “cartography” and its derivatives) used here and elsewhere in the project are intended according to common sense and not in the stricter epistemological sense ( Tambassi, 2021, 4–6). For this reason, issues related to the manyfold aspects of geography, including, for example, human geography are not covered. 

Geographical names, historical characters, mythological characters, when possible, have been connected to other online resources, such as geographical gazeteers ( Pleiades), later travellers’accounts (Travelogues), ancient sources collections ( Topostext), digital dataset of Greek myth ( Manto), monuments databases (such as Kastra), and/or websites of other relevant projects.

A separate file sheet has been prepared for all the geographical annotations present on the maps and the links to the relevant resources mentioned above. 

## Choice of the tool

When it came to choosing the tool to organize the data and create the edition, there were multiple factors kept into consideration. 

The project is relatively short (three years) and given the nature of the Individual Marie Curie Fellowships, it was not run by a multidisciplinary team but by one scholar alone. Even considering the relative simplicity of TEI and its annotation system, the lack of an interest in creating a traditional philological apparatus and the heavy size of the commentary compared to the text it refers to did not encourage in that direction. Furthermore, the beginnings of our research on Buondelmonti’s text date back to a couple of decades and the results were stored in Word files, some of which had already been published in a traditional way ( Bessi, 2009; Bessi, 2014). It was important therefore to find a system that allowed the reuse of these legacy data without requiring a too complex transition.

Planning, as mentioned above, to keep the project open to future expansions both in the content of the commentary and in the layers of analysis (such as, for example, philological or geographical), we were also interested in a tool that could support a modular structure of the data and could offer the possibility to re-arrange and expand them without compromising their initial organization.

Both theorical and practical reasons directed our choice.

First, on a more general methodological ground, the multi-disciplinary nature of our subject is such that it calls for an open-ended model. Of course, this often is an aspect we may find in most digital products, due to their dynamic, ever-evolving nature. So, the publication in a sense marks the start, rather than the end of the research process, as it provides not only its specific results, but also tools and models which can be reused by its scholarly audience for other purposes.

Yet, in this specific case we are approaching a subject which intrinsically is composite, ranging from geography to ethnography, from history to mythology, from archaeology to philology, *etc*.; and potentially involves a lot of different directions of specialization. Further, this text has never been made available with its text and a reliable commented translation, so that MapAeg here is really laying the ground for more future constructions.

Of course, exploring some of these directions of expansion would involve a wider scholarly team, and the definition and integration of totally new models. This implies that whatever our starting model, it should deal with the fact that it might possibly be expanded and specialized in multiple, completely different directions. Also, on the practical side, such an expansion should be possible without affecting the existing data and their models, together with the software based on them; and be based on a content creation procedure as friendly as possible, to allow many different specialists to contribute to the project, even without a specific IT background. On this way, such projects might even be open to controlled expansions to community-wide contributions, especially because many of their details are locally scattered across a very wide and relatively isolated ground, and this calls for an easy content creation process at both the expansion and creation stages.

Yet, at the same time, we also need to be able to ensure the standard, specialized outputs expected by most scholarly communities, thus granting interoperability and sustainability, like Text Encoding Initiative (TEI) exports, or even Resource Description Framework (RDF) graphs or service endpoints. We would thus need a higher level of abstraction in our modeling, so that it can be the shared source of many different, virtually unlimited, and highly specialized outputs.

Finally, in a sense this requirement for such an open-ended, dynamic modeling not only refers to the future, but also to the past; that's because MapAeg was modeled as a digital product only after having already produced a lot of legacy material in the form of traditional word processing documents. So, here our models must be able to deal with any future expansion; but also be the target of an automatic import process, which not only consists in a format conversion, but requires remodeling the original content.

So, it is right the consideration of all these heterogeneous and even conflicting requirements which led us to the choice of a higher-abstraction level data and software architecture, based on modularity, reuse and dynamic composition, providing full graphical user interfaces directly exposed to the web and targeting a centralized database. This would be a sort of content creation and integration hub, placed at the intersection between import and export paths, and linked to any third-party resources. All this is provided by the open-source Cadmus content creation framework.

To test the tool, we chose to apply it on the paragraph on Corfu: not only the description of this island and the related map show up as first in the *Liber*, which at least in the initial paragraphs reflects the west to east navigation of the author, but it also happens to be material that had been already published in a traditional format few years ago ( Bessi, 2014). Furthermore, given the size and the importance of this island since antiquity up until to Buondelmonti’s own time, both the description and the map are rich in details, and the abundance of this material offers therefore a good testing ground for the most complex instances.

## Leveraging the Cadmus tool

In the context of this paper, rather than presenting in detail frameworks like Cadmus, which are partially covered by some literature and more online resources ^1^, we’ll try to show how they can effectively be used in a larger data flow, where modeling has a paramount importance for both legacy data recovery and new content creation.

To this end, this portion of the paper will be organized in three main sections, so to emphasize how the content creation system used here is placed at the center of a flow including import and export capabilities, while acting as a sort of data hub in integrating external resources and services. By following a full flow, we will thus start from legacy data recovery and import, continue with its modeling and editing, and finish with some examples of its exports.

### Recovering legacy

In this project, most of the start material technically is legacy, as it is represented by Word documents. Each of them includes both the Latin text and its English translation, usually accompanied by footnotes. These may belong to different categories and include references to ancient or modern sources.

Given the constraints imposed by this starting point, and the requirement to preserve the original design and workflow as far as possible, we must be able to recover all these data and add only a minimalist structure to them, at least capable of separating the different texts and notes, so to combine them later in a more interactive presentation.

So, the main issue here is dealing with a typographically structured, continuous text document, which should be converted into some semantic structure for further expansion and processing. More generally, a set of unstructured Word documents happens to be a very common scenario when dealing with legacy data, either digitally produced or acquired ( *e.*g *.,* from paper editions). Of course, documents might present totally different layouts and structure, and we might even deal with other formats, ranging from plain or rich texts to spreadsheets and databases, all being either standard or proprietary. In some extreme cases, it might even happen that the text encoding itself refers to obsolete standards, or, even worse, to totally proprietary solutions.

Yet, there are a lot of precious works which could be recovered from such legacy state into a modern, standard technology, so that it can be returned to the community of scholars and more generic users. Thus, dealing with projects which present similar challenges can also be the occasion for attempting to design and implement solutions capable of being reused.

So, the first task in our list is represented by recovering and remodeling content from legacy Word documents. To this end, we use a tool of mine (Proteus) which has been already designed and repeatedly applied in real-world scenarios to recover data from many different sources, adopting a unified model for their representation as the input to the conversion process. So, here we will just introduce its essential traits to show its role in the general workflow of this paradigmatic project.

Essentially, Proteus works by dissecting each input format, whatever it is, into a sequence of so-called *entries*. For instance, let us consider the case of a rich text like a Word document: we could extract its content as a long sequence of such entries, each representing a portion of the text, or some simple formatting information ( *eg.* bold or color), or more complex layout commands ( *eg.* the various properties of paragraphs, footnotes, etc.).

**Figure 1.**
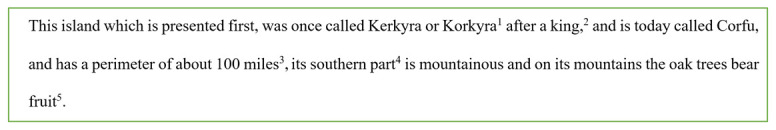
A sample English paragraph.

So, let us consider the first English paragraph of a sample document about Kerkyra ( Figure 1): it is a simple, unstructured text, with some footnote references.

**Figure 2.**
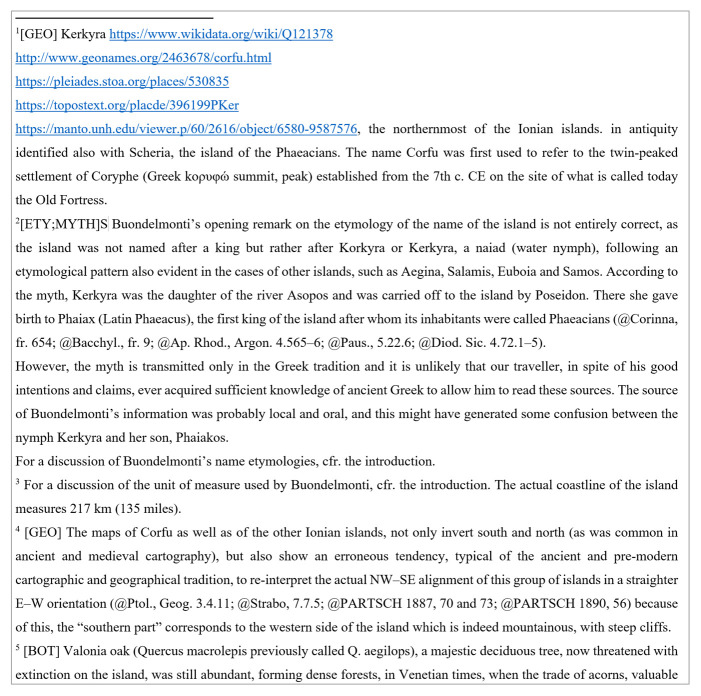
Footnotes related to the sample text of Figure 1.

The corresponding notes ( Figure 2) are just free text, except for a few markers the author has chosen to add to provide a minimalist structure; for instance, you can see categories between square brackets, or at-marks before an ancient or modern reference. For the rest, this is just an ordinary word processor document.

**Figure 3.**
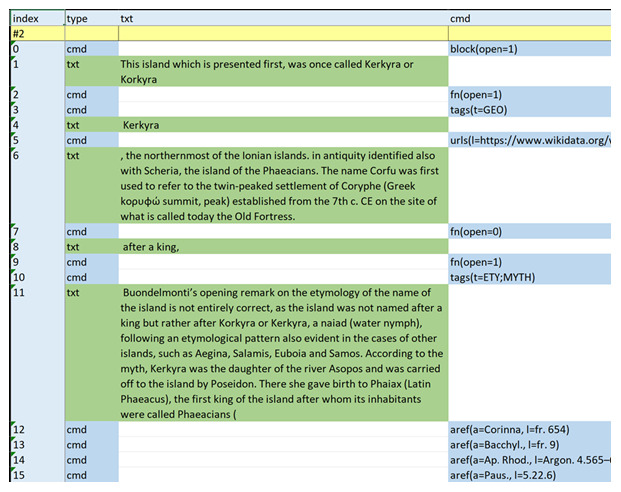
A portion of the diagnostic dump of the entries parsed from text and footnotes.

Now, this text is parsed by the Proteus system components and eventually dumped into an Excel file, to provide a diagnostic view of the results ( Figure 3). Just like the model used here, reading this dump is quite easy: you go across the rows, from top to bottom; each row corresponds to an entry. According to its type, the various columns of the row are filled with data and colors.

Thus, row 0 is just a command entry starting a block of text (=paragraph); a text entry follows, with a portion of the text. Then, another command follows, this time representing the opening of a footnote; another command (at index 3) is the result of the parser having detected the initial text of the footnote as a set of categories. The outcome is a tags command, whose arguments list one or more tags as extracted from the text within square brackets.

Then, other text rows follow, variously interrupted by non-textual entries as extracted by the parser: reference URIs, footnotes end and start, other tags, ancient documental references, etc.

So, in the end the structure of the input text has been flattened into this long list of atomic entries, each having its own type and meaning, while still belonging to a uniform data model.

This is a minimal example, extracted from a DOCX parser; but it should be easy to imagine how the same modeling of input data might be used to represent not only other rich-text formats, but also plain texts where implicit typographic or semantic information can be extracted by inspecting their content, or even totally different data sources, like spreadsheets or databases: we just provide the importer software with a sequence of ordered entries representing unstructured or low-structured data, mostly in a human-friendly, text-based format; and let it work out the target models we want to extract from it.

To perform its transformations, Proteus leverages a composable pipeline, driven by an external configuration document. This defines which plugin components to use in which order, and how to configure them. Several plugins are already provided by the system, and cover most of the usual requirements; anyway, users are free to add their own with specialized logic.

Plugin components essentially belong to the following categories:

- *entry readers*, which read a data source ( *eg.* DOCX, XLSX, TXT, CSV…) emitting entries.
- *entry set readers*, which split a continuous list of entries into sets, corresponding to some division meaningful for the source being handled. These sets represent self-contained work units, more manageable than the full, continuous list of entries. For instance, should the source be a dictionary, a set would be a single entry in it. In the case of this project, where the text flow alternates Latin and English paragraphs, the entry is a single paragraph. In Figure 3, the yellow line at the top represents the start of a set with its ordinal number ( #2 ).
- *entry filters*, which modify the received list of entries in any way useful for further processing. A special type of entry filters handles escapes, *i.e.* any sequence of character(s) having a special metatextual meaning in the text to be converted. Such filters include one or more sub-components, named escape decoders, capable of matching escape text and decoding into further entries. In our project, escapes are text like category tags, URIs, or references.
- *entry region detectors*, which detect semantically meaningful regions inside a set of entries. These regions can have any extent, and freely nest or overlap. For instance, in a dictionary, regions might be lemma, etymology, translations, examples, inflection, or any other semantically defined field in its text. In our project, regions are much less granular, and just define the topmost text areas: paragraphs with their language and footnotes.
- *entry region filters*, which may variously postprocess detected regions.
- *entry region parsers*, which parse entry regions to build some other digital representation from them, including dumping them for diagnostic purposes. Each parser is usually applicable to one or more specific regions.

By combining all these components together, you can define highly modular and reusable transformations to recover legacy data while remodeling it, whatever their digital encoding and format ^2^.

In the context of MapAeg, this architecture has the additional benefit of allowing variations in the input flow: for instance, this project also includes a group of more recent documents produced by the author, still lacking a systematic recognition of all the semantically relevant entities cited in them (toponyms, anthroponyms, etc.). Even though we cannot revolutionize the original workflow as conceived by the author, we can still support her work by introducing a NER service in it. In this case, we can use Proteus to extract text from DOCX and adjust it in the format required by this service; then run the service and remodel its output in the same way. Just like we introduce entries for categories, references, and the like, we can introduce entries for toponyms, anthroponyms, etc.

This will have the effect of branching the original workflow: on the original side we will have a direct extraction of entries from DOCX; on the new side, DOCX will be extracted into an intermediate format (essentially XML), passed through the NER service, and finally merged with the same entries-based model already in use. In both cases, the final outcome becoming the input for the importer component will be equal, so that whether a source followed one of the two branches is totally irrelevant for the system.

Thus, the application of a system independently developed for similar problems turns out to be effective also at this lower scale, besides providing a paradigmatic case study to illustrate its methodological tenets. In fact, it is easy to understand how this is not only a digital format conversion, but a deeper remodeling of a data source, from its typographic structure to a semantic one. It is right this conversion which introduces us to the next step in our workflow.

### Importing and editing data

As we have seen, preserving a base compatibility with the original author’s approach and her existing materials is here of paramount importance. Yet, the nature of these data itself claims for at least the potential of a much more granular structure, together with the ability to expand and specialize them even with external contributions.

In fact, even if we just start with a rather traditional comment on a text, it is right the genre of the text itself which allows for a lot of very different and more specialized expansions, covering domains like history, geography, mythology, literature, archaeology, ethnography; and for all these new domains, we know nothing about the models we might want to design in the future. So, rather than recreating new editions from scratch, we should be able to create new contents on top of the existing data, whatever their modeling, and allow contributors to do this concurrently. In this context, a primary concern would be designing reusable software tools to support highly structured and modular content creation, so that their models are open to expansion in any direction by any number of team members.

Another related point is represented by ease of use, as in the approach adopted here this becomes a consequence of a higher level of abstraction: scholars should be able to focus on modeling their own knowledge domain and create their data accordingly, without being too much constrained into the cage of physical models. This not only means making them able to do this without specific IT skills; but also letting them define their objects with higher levels of freedom, without having to worry about technical details.

Often, a lot of effort is put in finding out more or less creative ways for hijacking the technology you want to target so that your specific model can fit into it. Sure, the purpose of this effort is right to attain that level of standardization which allows your digital product to successfully interoperate with other resources; but in some circumstances, it may happen that the adaptation effort is even bigger than the scholarly work itself. At that point, the target technology starts looking more a problem than a solution, which may even drive less digitally versed scholars to leave this path, and rather continue with traditional approaches.

On the other hand, a digital project is as relevant and long-lived as it is interoperable; data should not be siloed into some proprietary container nor be created for the only purpose of presenting it in some web application. Rather, we should at least be able to export data, and even their potential future extensions, into standard technologies representing the current state of the art in publishing and sharing them, like TEI documents, RDF-based semantic web models and API services, and any other publication method.

Finally, as already seen for newly created documents, it would be desirable to foster integration since the content creation stage, by including third party services and resources, like International Image Interoperability Framework (IIIF) images, geographic gazetteers, ontologies, generic or specialized data repositories, etc.; or even before it, at the conversion level, for instance to provide automated ways of easing the detection process for words like anthroponyms and toponyms, typically leveraging named entity recognition (NER) systems based on geographical gazetteers.

Of course, all this is far from being feasible with a simple set of Word documents, even though the structure of their derived data is intentionally kept at a relatively low level. Also, Digital Humanities projects mostly gain additional value in designing and implementing methods and tools at a higher level of abstraction, so that they can be applied to other projects as well, without forcing scholars to reinvent the wheel for each of them. So, devoting more resources to methodological aspects and tooling, even when this might seem an overkill for a specific scenario, can be much more rewarding, not only to be able to fulfill the requirements, but also for contributing to more widely shared solutions with paradigmatic case studies.

In this scenario, the solution provided as a sort of hub where content is both imported and edited, external resources can be leveraged, and virtually any kind of output can be generated, is represented by my Cadmus framework. This framework has been primarily designed to face potential issues with the creation of highly structured and complex content, whatever its type, by raising the abstraction level so that physical models can be superseded by logical models, and their looser connection with a specific serialization technology can ensure more freedom in their design. So, this is not just a way for creating text-based content, like a digital edition; but rather a true, general-purpose content editor, where any type of data can be modeled, and designed in such a way that it can be used as a framework for totally different projects. This is intended to foster collaboration and sharing, while providing an economical way of creating highly specialized content in a distributed and layered editing system, without having to reinvent it for each new project.

In fact, a key concept in Cadmus is right *reuse*, of both data models and their editor user interfaces (UI). Data models are composite and dynamic, open to change and expansion; and each of them comes with its own editing UI, so that the resulting editor is built by composition, too. In turn, the key to reuse is *modularity*. As for LEGO bricks, we can assemble unlimited buildings using the same components; and this requires partitioning models into smaller pieces, making all the problems look similar by raising the abstraction level.

In this architecture, you can think of core data as more or less complex objects, each having its own set of properties, which in turn can be other objects. These objects, named *parts*, are modeled so that each of them is limited to the simplest and smallest data domain which can be usefully represented as a reusable, independent building block. For instance, an object can be represented by a single historical datation with all its nuances; or by a set of categories, drawn from some external taxonomy; or by a set of keywords, with their language and group; or by a set of proper names, fully structured with their components and their metadata.

All these objects are then assembled together to represent a bigger data unit: for instance, a person record could be composed of a names part, collecting all his/her appellations; a datation part, locating that person in a time span; a categories part, tagging that person according to taxonomies defined by our project; and again, an events part, listing all the relevant events occurred in that person’s life, from birth to death; an external identifiers part, connecting that person to the codes used in other resources to identify him/her; and any other type of component useful to say something about that person.

You can thus imagine this composite record, named *item*, as a box capable of containing as many objects as you want, whatever their type. Whenever you want to say something more about that record, you just toss a new object into this box. The independent and relatively atomic nature of each object model grants the possibility of reusing them for boxes representing a lot of different things: for instance, most of the parts cited for the person example could be equally applied to a manuscript, an inscription, an archaeological artifact, a literary work, or immaterial things like concepts, narratological themes, linguistic items or any kind of entity.

In most cases, such object models are highly structured; for instance, the historical datation model includes two dozen properties, capable of representing nearly all the nuances you might want to use when defining a point or an interval on a timeline. Yet, this does not rule out the possibility of lower structure parts, like *e.g.,* a generic, free textual note: in this case, the whole model consists of a rich text (usually encoded as Markdown) and an optional tag for grouping or categorizing it. So, should you ever want to add some miscellaneous, free text note to a person, a manuscript, an inscription, a dictionary lemma, or any other item type, you would just have to add a note object in its box.

Now, it should be emphasized that the same modularity of this data architecture is found also in the software: each of these parts not only has its own, totally custom data model; but also has its own, totally custom web UI used to edit it in a user-friendly environment. So, not only data models, but also its editor are built by composition; the editor just composes all the parts together, orchestrated by the Cadmus infrastructure. Every part editor can be as simple as a web form, or as complex as a fully self-contained web application; and often it can leverage external services, like IIIF, data lookup services, and any type of internal or external resource.

So, in this architecture you are totally free to design any object model you want, whatever its complexity and structure, without being constrained by any specific serialization technology. Even if Cadmus provides several stock parts, every project often adds its own, specialized ones, which are freely mixed with the others ^3^. Also, you effectively deal with dynamic, composable and open models, as far as your records are not directly these objects, but rather the boxes which include any number and type of them. The item’s model is just the sum of the parts it contains, which means that it changes whenever you add or remove any of them; and this can be done indefinitely, without affecting existing data.

In the same spirit, also all the taxonomies used by parts and their editors are totally up to the project using Cadmus, either they are just flat lists, or hierarchical structures. Usually most of them are defined and imported once from JavaScript Object Notation (JSON) documents; but the editor infrastructure allows editing them directly within the UI. Taxonomies are effectively part of the profile defining the whole data architecture of a project, which also includes the system the items defined, the parts used by each of them, and even the components used to provide mock data to play with a newly assembled editor.

As a final remark, more directly connected to the MapAeg project, let us consider how text and its metadata are handled in Cadmus. The higher abstraction level adopted by it makes it possible to use the same architecture illustrated for any other content type. In fact, text here is just an object, like any other piece of data. When you put this object part inside the item’s box, this item becomes a text item just by virtue of this action.

According to the atomic and independent nature of Cadmus parts, the text part has a single responsibility: including plain text. In turn, all the annotations on it (the equivalent of tags in XML) are represented by other objects, each having its own model specialized for an annotation type. Such objects link their annotations to the base text object, so that they work as layers on top of it: for instance, you might have a critical apparatus layer, an orthography layer, a paleography layer, a prosopography layer, and as many other layers as you require for your specialized annotations.

Such an architecture works best whenever metatextual data are rather heterogeneous, and/or can grow up to a point where they by far outnumber textual data; and this may happen even with very short texts. For instance, let us consider even a couple of words in an inscription text, like *que bixit* (= *quae vixit*, referred to a female deceased name), having a ligature between the final *-e* and the initial *-b*: here, we might want to annotate that *que* is a non-standard orthography for *quae*, and *bixit* for *vixit*; further, we might also want to add more data about these orthographies, to link them to the linguistic phenomena underlying them (monophthongization of *ae*, spirantization of *b* converging with *v*, so that hypercorrected orthographies like this arise). Additionally, we should also annotate the ligature between the letters of these words. So, even with just a couple of words we are facing the problem of having to add complex metadata ( *e.g.,* not just the standard orthography, but also a fully structured and detailed linguistic classification of the underlying phenomena), belonging to totally different knowledge domains (like a ligature *versus*. linguistic data), and yet overlapping at the same document position, with different levels of granularity. Usually, in XML-based solutions we can perfectly accumulate such metadata using nesting, milestones, or adding new attributes; but this implies changing the existing structure whenever we add a new annotation, and complicating it further, up to a point where it is no longer feasible, or hits the overlap limit. Also, this mixes two different knowledge domains into a unique structure, and constraints their respective models as far as they must be expressed in terms of tags and attributes and fit into the puzzle of the existing markup.

**Figure 4.**
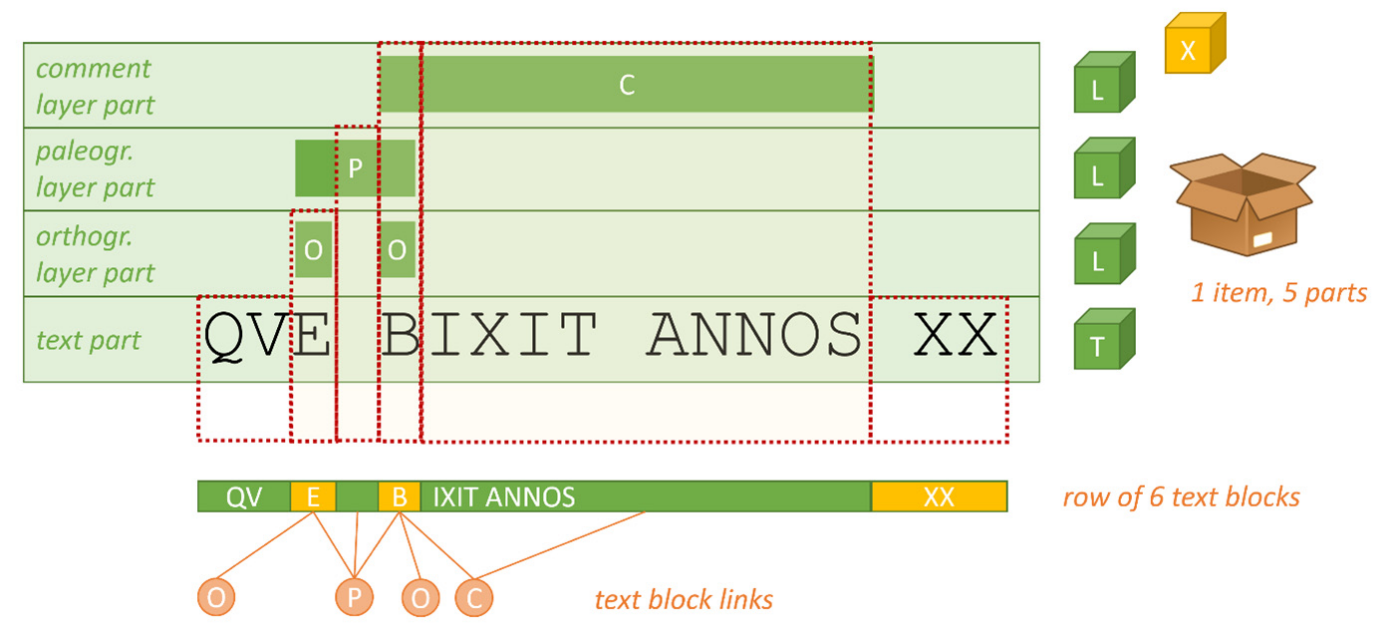
Flattening layered text for export.

Within the Cadmus architecture instead ( Figure 4), these data get distributed into different, independently designed objects: the inscription itself is a box (an item), while its text, orthographic annotations with their linguistic modeling, and paleographic annotations are all separate objects, acting as layers, just added into the same box.

So, this layered text architecture allows for an unlimited expansion of annotations because each type is isolated in its own layer object, and freely modeled according to its subject, without any constraints from the context. Also, the corresponding target text is not affected by the addition of any of these layers, because these are all independent objects, freely added and removed from the same box.

In scenarios where you have to fit too many different metadata on top a unique hierarchical structure represented by text, typical when dealing with XML, this provides an alternative solution which removes most of the issues arising from it: annotation models are free to be designed in total isolation, without being constrained by the requirements of fitting them into the mosaic of other annotations, nor limited by a tag or attribute based implementation. Also, adding any annotation has no effect at all on the existing ones, whereas in XML the unique underlying structure changes at any new addition; nor there are limitations coming from the physical model, like overlap.

Finally, another advantage is that the user experience is dramatically simplified, so that users can literally know nothing about technologies like XML: all what they do is selecting the text they want to annotate and click a button, to get into the editing UI for the chosen annotation type. As remarked above, each object not only has its own model, but also its own editing UI; so here too the layers editor is just a composition of part editors ^4^.

Of course, we can continue on this path indefinitely: the same inscription box could contain new objects, representing other textual layers, but also non-textual data at all, like the physical support description; its archaeological context; its GIS-powered geographical location; a historical commentary, and any other layer: these is no limit to the expansion of the model.

In the end, this brings together non textual data, textual data, and meta textual data, in an open-ended, modular architecture, with a uniform, abstract architecture for all, in what becomes a sort of data integration and editing hub, at the center of a wider data flow.

Also, the modular nature of this architecture can lay the foundation of a wider cooperation among scholarly projects, as building by composition implies confronting with others’ models, and often reusing a lot of parts created by a project into a totally different one. Thus, as any project designs its own models and UIs, these converge into a sort of shared catalog, so that other projects can take advantage of it and even build a full-fledged editor by just assembling existing parts. This not only eases the creation of the editing system, but also promotes a virtuous circle towards shared models for various scholarly domains.

### Exporting data

In the end, Cadmus is just a content creation system, placed at the center of a potentially wider data flow, where content enters from one side, eventually remodeled as required, and exits from another one, remodeled to fit the addressed technology.

Once content is there, we can of course export it into other digital formats; you might even treat Cadmus just as a content creation tool between optional import and export stages and dispose of it and its database once you get its output in the desired target technology.

Additionally, as a further level of integration, in the middle of this flow (the editor) we can also add any type of external resources, typically but not exclusively in the form of services consumed by the editing system (think *eg.* of IIIF for images, IconClass for their features, VIAF for authority files, or various semantic web ontologies); this provides a centralized data hub with web-based concurrent access, open to team or even community work, and backed by a full layered system targeting a set of databases.

This hub may include just newly created data, or legacy data imported and remodeled for it, or any mixture of both; in the end, everything that enters it gets into a uniform data architecture with open-ended, composite models, so whatever its origin it looks the same to users, just because it effectively is the same: any type of content here has been leveled up to the desired set of abstract models. This way, old and new content can be merged and expanded at will, for both their quantity (by adding new data of the same type) and quality (by adding new types on top of the existing ones).

On the other side of the flow, any output could be provided as a service or as a data export. For instance, two of the most popular export options are represented by TEI (for those projects having text) and RDF (for semantic web), so we can use them as real-world examples ^5^.

Here, we should first emphasize that for both these exports the data source is exactly the same, and is created once *via* a web GUI, by people who can be totally unaware of either XML and RDF; all what they need to do is filling more or less complex web forms. So, this allows scholars focusing on their own subject matter, without requiring several IT skills to model and create them. This provides the benefits offered by such technologies, without most of the issues arising from having to deal with them at a lower abstraction level: instead of users editing XML or RDF, you just have software creating them from a user-defined database, with several reusable approaches accoding to the target ^6^. So, following our example, let us briefly consider the TEI case.

### TEI export

In general, a typical export is not just a digital format conversion, but rather a remodeling process which transforms a structure into another. This is also the case with TEI output.

As illustrated above, Cadmus handles text just like any other data, *i.e*. as objects. While in TEI the text is typically divided into sections (using elements like div ), here it is divided into items, the “boxes” including any type of object. Among these objects, one represents the text itself, while others may represent specialized layers of annotations on top of it, like critical apparatus, paleography, orthography, linguistics, or any other layer, whatever its knowledge domain.

As remarked above, in this architecture there is no limit to the annotations, and each type of annotation is isolated in its own layer and modeled on its own, as an object with any level of complexity. Adding new annotations, or new types of annotations, has no impact at all; either on the target text, or in any of the existing annotations in their layers. Also, their extent is not constrained by overlap limits: two annotations in different layers can freely nest or overlap.

Thus, this layered text architecture is much more scalable and open in comparison with an ordinary TEI document, where a well-known constraint is represented by the fact that all data rest on a unique, tree-shaped structure, where no overlap is possible. In XML, adding new metadata on top of a text implies the modification of this unique structure, by adding new elements and/or attributes, often resulting in more complex nesting. Also, this indirectly constrains the model underlying the added annotation, as it must be implemented as a set of tags and/or attributes; and all these must fit the existing structure, which often targets completely different knowledge domains.

At any rate, adding new metadata in XML is feasible only as far as complexity remains below a certain threshold; and it’s not possible to overcome the overlap limit. When this happens, the only practical solution is standoff notation ^7^, which effectively multiplies the trees by providing several documents with annotations variously linked to the one with the “base” text. This solution anyway proves very difficult to handle by hand, and in most cases, users need some *ad-hoc* software tool to handle it. Yet, as here we are generating TEI in a totally automatic way, this poses no issues as Cadmus export components are already in charge of this task. In a sense, the standoff notation structure can be easily compared to the Cadmus architecture: there, you have a main document and various satellite documents with further annotations linked to it; here, we have an item (a box) including several objects, one representing the “base” text and others representing layers of annotations on top of it.

Of course, the main practical issue here is that Cadmus is has multi-layer architecture, where several annotations freely overlap at variable granularity levels. It may well happen that one layer selects a single character of a word, while another selects three words including that same word. We thus need to "flatten" these layers into a single sequence of characters, corresponding to the base text to be annotated.

The key to this flattening process is merging all the layers selections into one, via a model based on *text blocks*. A text block is an arbitrary span of text, linked to any number of annotation layers.

**Figure 5.**
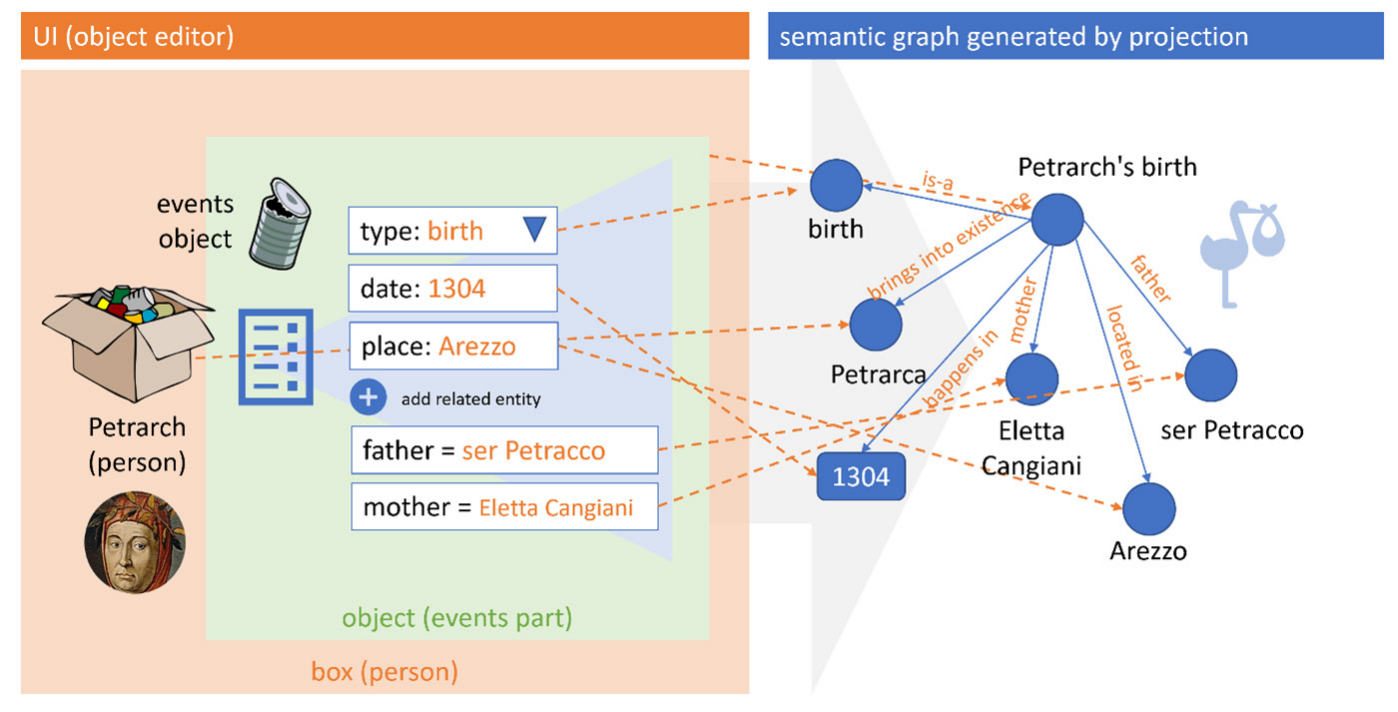
Mapping a subset of a data part into an RDF-like graph.

For instance, say you have a simple line of text like the one in Figure 5, where different portions of it are annotated at different levels (each represented by a letter: C =comment, P =paleographic annotation, O =orthographic annotation). In the usual Cadmus metaphor, this means that your item box contains at least four parts: one for the text, and other three for the layers.

Here, the orthographic annotation ( *que* for *quae*; *bixit* for *vixit*) rests at the maximum level of granularity (the character), and so it is for the paleographic annotation ( *e.g.,* a graphical connection between *E* and *B*, including the mid space). The generic comment annotation instead covers two words, showing how *bixit annos* uses the accusative rather than the more usual ablative. Each annotation has its own extent, at different granularity levels and most of these spans overlap (but this is not an issue, as each one rests on its own layer).

Now, when flattening these layers into a single sequence, the text gets split into blocks defined as the maximum extent of characters linked to the same layers. So, the resulting blocks will be six: *qu|e| |b|ixit annos| XX*. Each of these blocks is linked to several layer annotations; so, it’s now easy for an exporter component to handle the flattened output as a text segment connected to different annotations. Additionally, the exporter allows to pick only the layers you desire, so that only the subset of metadata you want can get into TEI.

As a further benefit, in this approach we are not required to split the text document with a predefined level of granularity. This is usually required in TEI stand-off, where you provide a text before annotating it. For instance, if you are going to annotate graphical words, and nothing below this level, you will just mechanically wrap each sequence of characters delimited by whitespace into some element, assigning to each a unique ID, like this ( *Catull*. 2,1) ^8^: <l><w xml:id="w1">passer</w>, <w xml:id="w2">deliciae</w> <w xml:id="w3">meae</w><w xml:id="w4">puellae</w></l> . This is of course redundant, as not all the w elements (and their IDs) will be effectively used as target of linked annotations; but you need to systematically wrap all the words, as you can't know in advance which words will be annotated. Also, and more important, this limits the annotation to the chosen granularity level (here the graphical word). Should you want to annotate a single syllable, or a single letter, this would require further interventions.

In Cadmus instead, TEI is just one of the many outputs which can be generated from the objects in the database. So, not only does this mean that we can regenerate the full TEI documents at any time; but also that, when generating it, we can rely on a sort of “snapshot”, where we know in advance which portion of text will get which annotations. We can thus wrap portions of text of variable granularity, without having to stick to a predefined unit. Just like in Cadmus you annotate text by selecting any portion of it, from a single character to several lines, in TEI we will wrap different spans of text corresponding to such selections.

Given that Cadmus architecture is uniform, whatever its content types, this also means that the above export procedure can be systematically reused across different projects, even though we are free to customize it by replacing some terminal components with others in the export pipeline. Also, if we add new layers, we can just regenerate the whole set of TEI documents.

### Exporting RDF

The second example chosen to illustrate the flexibility of this sort of data editor hub is represented by another, even deeper remodeling into an RDF-like graph ^9^, while also integrating it in the context of the editor.

In fact, one of the reasons for this export was also the requirement to express complex relationships among the entities represented by Cadmus models (the boxes and their objects). As we have seen, each object has its own independent model, and it’s right this independent nature which grants its reusability in different contexts. These models may well include relationships with other entities; but usually these are limited to those relationships considered as intrinsic by their nature in our modeling.

For instance, say, we have a project dealing with entities like persons, manuscripts, and letters. We might certainly consider the author of each letter as an intrinsic property of its model, as in our scenario no letter is without an author, which brings it into existence. Yet, we might also want to represent more accidental relationships: for instance, a person may commission a manuscript; another one, write it; another one, decorate it; another one, comment it; then, the first person may write a letter, attaching the manuscript to it for sending it to again another person. Of course, all these events are rather ephemeral and virtually unlimited; and they would not properly fit any of the models for persons, manuscripts, or letters without polluting their model.

So, to represent these relationships while still preserving the independence of each object model, an RDF-graph like model is used. Its nodes and their connections are generated in a totally automatic way, *via* a set of external mapping rules. These rules can match any portion of an item or part by variously filtering them, and then project any subset of their data into a graph database as nodes and triples. Also, each single projected node or triple keeps track of its origin, so that whenever users update any object in the Cadmus database, all the matching projection rules are executed again for it; the resulting sub-graph is then merged into the graph database, by properly adding, updating or removing data. This way, users just continue entering data in the editor UI, without even noticing that at each data entry some rules step in to build and update the graph derived from it.

For instance, say you are editing a part representing any number of generic events: these might well be the events happened to a person, or to a manuscript or a letter. Focusing on a person’s bio ( *e.g*. Petrarch), the first event can be a birth: so, in the Cadmus editor UI the user picks it from the events type list ^10^; then, optionally a datation or a place can be inserted ^11^; and eventually any number of directly related entities, like a father or a mother (in turn, these can be other person items, or just some external entities).

**Figure 6.**
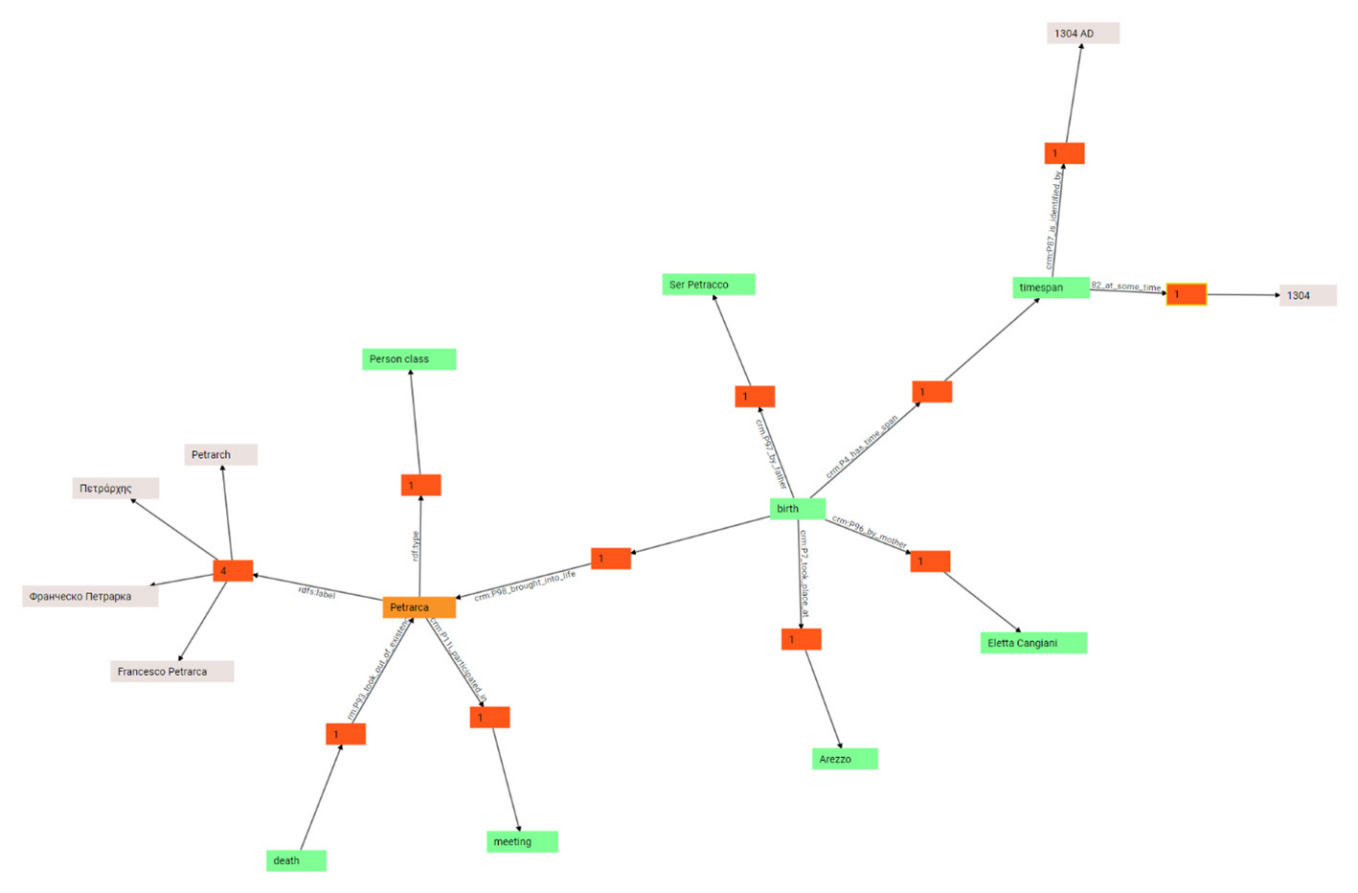
Interactively walking the graph in Cadmus UI.

So, in the end the user has just filled in a form to represent one of the events in a person’s bio. In Cadmus terms, the person is an item (the box), and the events list a part (an object in that box). This is enough to trigger several mapping rules, which project a subset of the entered data into the graph. So, if we chose CIDOC-CRM (CIDOC Conceptual Reference Model) as our target ontology ( Figure 6), the birth event itself becomes a node ( Petrarch’s birth ), being of type ( is-a ) birth; the date becomes a timespan node, connected to the event’s node; the place a place node, connected to the same node; while a father nodes participates in the event, and a mother node brings Petrarch (yet another node, of type person) into existence ^12^.

Once the graph is in place, users can literally walk it interactively, right inside the editor, and manually edit it, for instance to add new specific nodes or triples. So, this becomes a complementary data set, which while mostly generated by projection can also gain its own role of primary content. Of course, it is easy to export such a graph into an RDF resource, to be connected to the semantic web cloud; and even more, we can even totally change the ontologies used as our targets by just changing the mapping rules and regenerating the whole graph.

Again, here users work at a higher level of abstraction, which allows them using more synthetic and human-friendly structures to represent complex data; yet this does not rule out the possibility of generating from them more machine-friendly structures, like the highly atomized graph encoding an event like Petrarch’s birth in the above sample.

**Figure 7.**
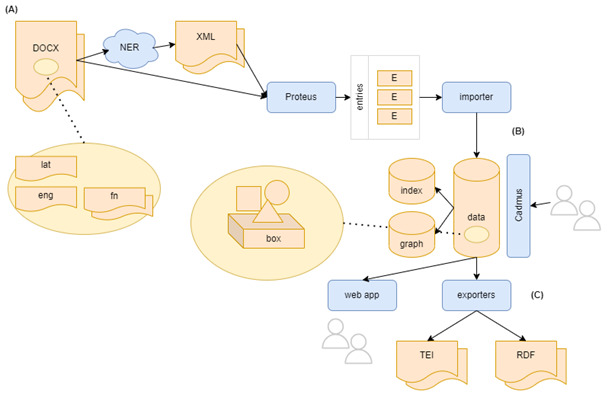
Essential aspects of the full data flow in MapAeg.

As in this case such structures are even directly editable, Cadmus provides a fully interactive UI to deal with them, by walking a graph from any node to any node, hiding or showing details as you go on ( Figure 7) ^13^. This UI provides an easy way of getting to the desired node for editing or inspecting it, while also visualizing the connections of a specific node.

As the graph may quickly grow up in size, it would be impractical to represent all the nodes and their links (edges) at once; the graph would be barely readable, overcrowded by a high number of overlapping shapes and lines. So, the solution adopted in the editor, where users may want to explore the relations starting from an object towards any other object, is displaying nodes and edges as you walk across the graph. Also, all the edges of the same type are initially grouped under a single graphical element (a properties group), with a number representing their count; this helps in reducing noise while exploring.

So, you start from a single node, and just see all its “outbound” ( *i.e.,* where this node is the subject) and “inbound” ( *i.e.* where this node is the object) links, grouped by type, with their counts. For instance, if the node has four labels in four different languages, you won’t see four links, but just a node representing their group. When you double click it, it will expand into those four links, each leading to another node. In the same way, you will be able to walk along all the links, from node to node, progressively unveiling the graph.

Additionally, a number of filters are available to be freely combined, so that you see only those links or nodes you are interested in. These filters vary according to the node selected while walking, and each node retains its own filtering state. Further, at any time you can go back from a node to the object which generated it via mapping, thus providing a fully integrated experience for jumping back and forth between different levels of abstraction in the same data architecture.

This not only provides the basis for representing complex, ephemeral relationships among Cadmus objects; but also allows integrating them by manually editing the resulting graph, and export fully compliant RDF for semantic web (and/or just a SPARQL endpoint), generated by the same objects at the source of TEI or any other kind of data export.

### Recap

**Figure 8.**
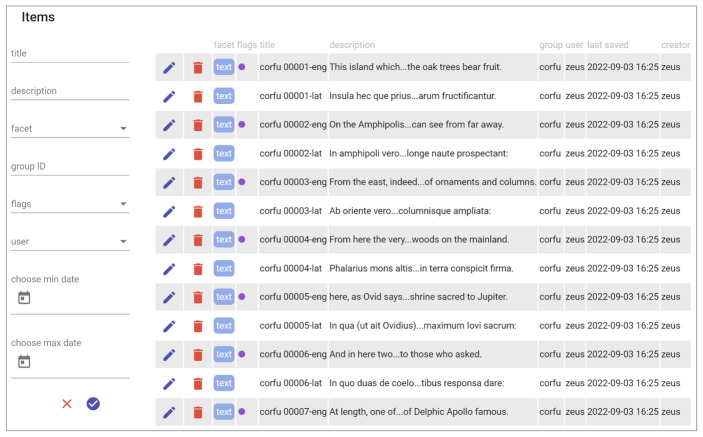
The first page of the list of Kerkyra items in Cadmus.

These examples about possible exports should be enough to represent the other side of the full data flow ( Figure 8), which starts from simple Word documents, and leads to many different semantically structured outputs, through a data editing hub which already provides a full standard database with all our data, whatever their origin.

In the case of MapAeg, the combination of legacy recovery, ease of use, and growth potential makes Cadmus a good choice even for a low-structure refactoring of the original data. Summarizing our flow, it all starts with pure legacy documents in Word format (DOCX), including alternating Latin and English paragraphs with footnotes (A). As illustrated above, these can take two paths which later converge into the same result: we can directly import them, or first have an intermediate NER service applied. In both cases, the next step is remodeling the input format, whatever it is, into a list of decoded entries, which gets processed by a Proteus pipeline to be transformed into a set of Cadmus models ( *i.e.* items and parts) via an importer.

Once data have entered the Cadmus hub (B), any number of users can edit them via its UI in a full-fledged, web-based environment, with a layered and distributed architecture powered by a set of underlying databases.

**Figure 9.**
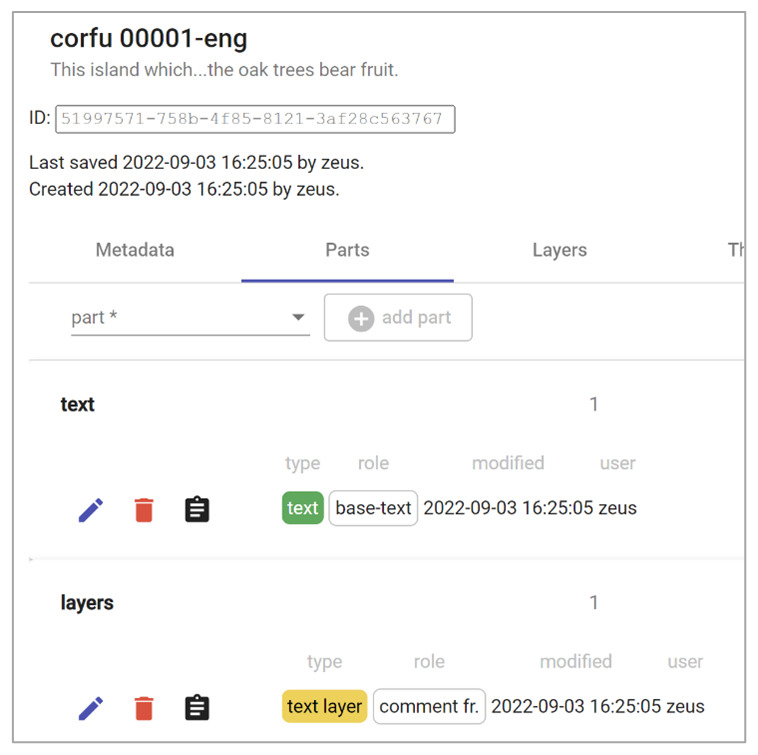
Editing an item’s parts.

At this stage, you can start working on data using the full-fledged UI provided by the editor. At start, you are presented with a list of Cadmus items, each corresponding to a paragraph of the source text ( Figure 9). Among other metadata, items may include several Boolean flags whose meaning and color is defined by the project’s profile: here you can see that English items are marked with such a flag, displayed as a small colored circle in the item’s row. Also, all the items belonging to the same text (here Kerkyra) belong to the same group, named corfu . Many filters are available to variously change the list’s content.

It is worth noting that the same UI can be used for any type of items, freely mixing them. In this case we just have text items, whose model is defined by the parts we insert in each of them; but nothing prevents us from adding new types at any time, whatever the direction of expansion or specialization we might take in the future: *e.g.*, mythological characters, literary texts, notable persons or places, historical facts, annotated maps, archaeological sites, castles, or any other topic. We will just have to design our models (or pick some from other Cadmus projects) and add them to the desired box.

**Figure 10.**
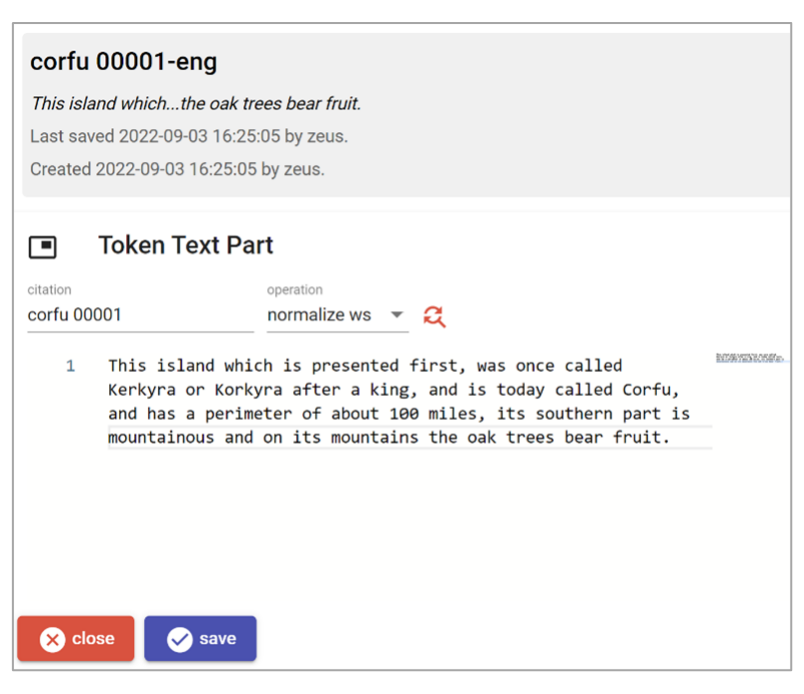
Editing an item’s text part.

To edit a model, we just click the pen button to open the item editor ( Figure 10). As boxes are all created equal, and their model is effectively defined by the objects they contain, the editing UI for them is unique. This includes a metadata section, with general item metadata; a list of the parts currently found in the item, plus a control to add new ones; a special section for those parts acting as textual layers; and other minor details ^14^.

**Figure 11.**
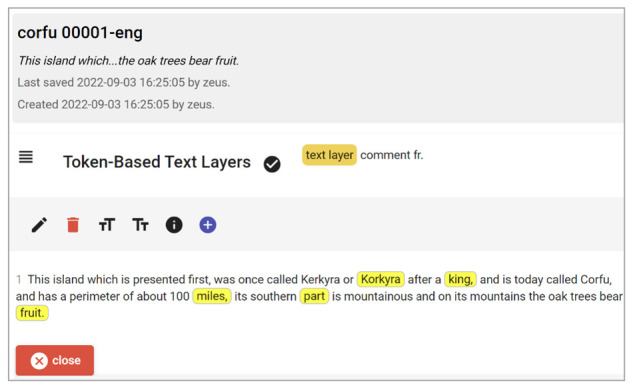
The shared portion of the layer part editor.

Editing a text object brings us to the corresponding UI, which is very simple as it must contain only plain text, with an optional citation ( Figure 11). In this case, the text has already been imported from the original Word document.

**Figure 12.**
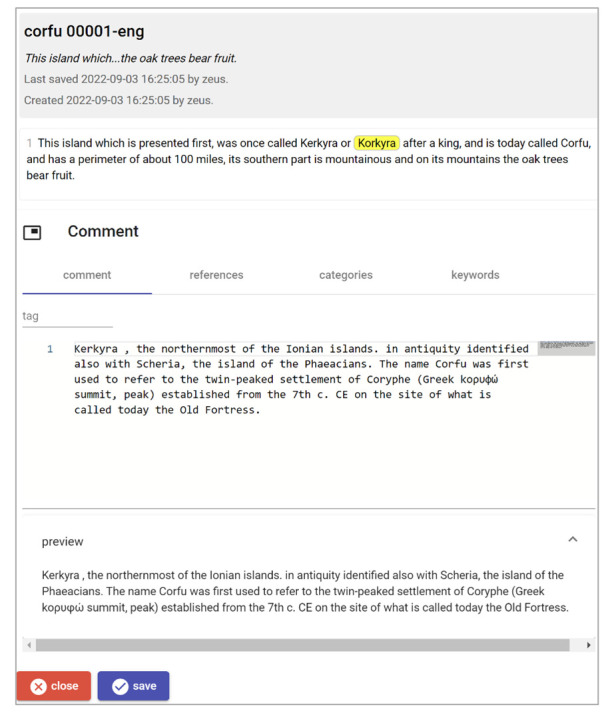
Editing a single comment annotation belonging to the comments layer part.

This is not the only object inside the box: we also have another layer object representing comments, here derived from the original Word document footnotes. The shared layer editor ( Figure 12) presents the base text with highlighted portions corresponding to the text having some annotation in this layer. Users can select any of their characters to edit the comment or select new spans of text (whatever their extent) and click the plus button to add a new annotation on this layer.

**Figure 13.**
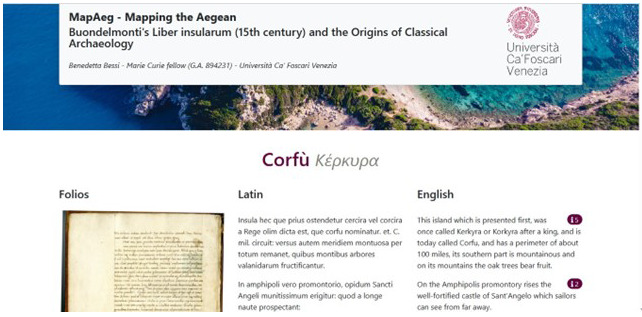
Digital edition frontend with facsimile, Latin transcription, and English translation.

In both cases, when editing an annotation, you are brought to the UI specialized for its own model, designed together with it ( Figure 13). The comments layer model has been designed as a sort of tradeoff part, to represent a traditional, free text comment, yet accompanied by some more structured resources, like references, categories, and keywords. Of course, nothing would prevent us to add more structured and highly specialized parts, either they are annotations on top of a text, or just correspond to non-textual data at all; but here the primary concern was ingesting the original Word documents into something more structured, easy to edit even in a concurrent way, and open to unlimited expansion.

So, here the comment layer brings into the database not only the footnotes text, but also those parts of it which have been extracted from the free text flow by means of the Proteus parsing stage, providing more granular data like categories, keywords, and references.

Once these data have entered the system, they can be edited at will just like we could also create new items directly in it, rather than by means of an import process; there is no difference between them, and no limit to their expansion.

Finally, at any time we can export (C) any subset of these data in standard technologies best suited to interoperability, like TEI or even RDF. The burden of this transformation is totally on software components, so that this effectively enables even users without any IT skills to just fill forms, and get TEI, RDF, or any other output without effort, while still having all the data available in a standard database. So, apart from standard exports and data or services offered for direct machine consumption, this also provides the backend for publishing the project as a standard full web application, targeting human users with a rich experience tailored to the desired audience. Whatever the presentations, the focus here is on content, and on the tools used to recover and create it, thus providing not only a finished digital product, but also a paradigmatic case study in content modeling and creation; so that the trip around Greek islands can also be the start of yet another journey in whatever realm you desire.

## Frontend output: an example of the digital edition

Although as we have just seen the flexibility and interoperability which characterizes Cadmus would support the use of the data in a variety of formats, as a first output, we created a web application which visualizes the data related to the textual description and the map of Corfu in line with one of the final goals of MapAeg which is meant to promote the importance of the Liber Insularum by allowing specialized and non-specialized readers to access its digital edition online.

**Figure 14.**
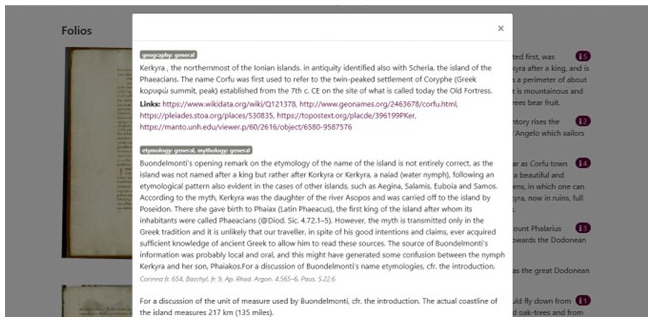
Popup with notes to the text.

This tester of what the full digital edition will look like, presents a facsimile of the Gennadios Library, Athens, ms. 71, folios 1 v., 2 r., 2 v.), transcription of the Latin text, English translation, and a commentary accessible on the right side. ( Figure 14).

The information present in the commentary is tagged according to various categories (etymology, geography, archaeology, settlements, monuments, mythology, history, botanics, text). The comments resemble traditional notes with text, bibliography and references to ancient texts but they are also linked to the other relevant online projects and sources already discussed above in this paper. ( Figure 14)

**Figure 15.**
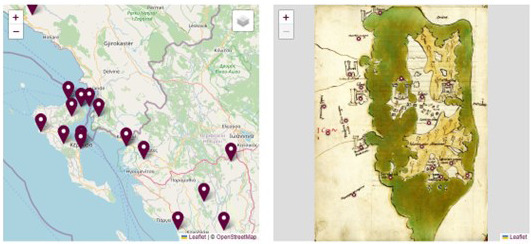
Juxtaposition of historical and contemporary map with pinned locations.

**Figure 16.**
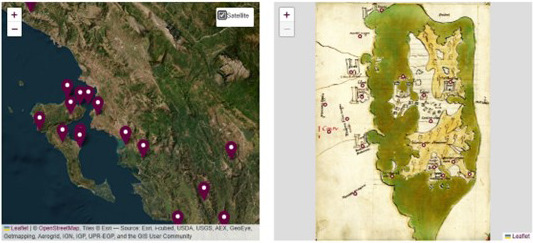
Same as above with satellite view.

The map which accompanies the textual description and is already visible in the facsimile reproduction, is presented again in an enlarged view and shown side by side with a contemporary map where corresponding places are geolocated ( Figure 15– Figure 16). Even if we are aware of the arbitrariness of juxtaposing representations reflecting such different theoretical conceptualizations of space and its representations, this practice presents itself as inevitable as it is also the case for all those digital projects in archaeology and other disciplines focusing on the ancient world where a comparison between ancient and modern geography is required ( Tambassi, 2018, 37).

**Figure 17.**
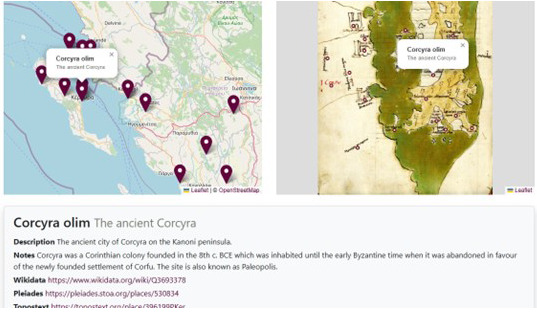
Detail with original toponym name in Latin, its English translation and entry with links to relevant projects.

By selecting one of the pins present on either the historical or the contemporary map, it is possible to access to a window with the transcription of the Latin annotation, its English translation, a short entry, and a link to the same digital resources also present in the comment to the text ( Figure 17).

## Conclusion

Although focusing on the example of a single island, we hope that this paper can provide a case study to show how a different methodological approach to the problem of creating complex and open digital content can also result in practical advantages, tested on a real-world project. Recovering legacy material, remodeling it into a much wider and generic data and software architecture, and still providing standard outputs ranging from web applications to API, TEI documents exports, or even semantic web data or endpoints, while still being open to virtually unlimited growth and community contributions, are all tasks accomplished in an abstract and modular environment, designed to be applicable to a high number of scholarly scenarios. We can thus meet both the requirements of a more demanding complexity and those of relatively loosely structured data, as derived from digital legacy content, while bringing them to the public of scholars and less specialized audiences in a web-based digital edition of the *Liber Insularum*.

## Ethics and consent

Ethical approval and consent were not required.

## Data Availability

All data underlying the results are available as part of the article and no additional source data are required. Source code available from: https://github.com/vedph/cadmus-migration/tree/master. Archived source code at time of publication: https://doi.org/10.5281/zenodo.10201220. License: Creative Commons Zero "No rights reserved" data waiver (CC0 1.0 Public domain dedication).
