# Supplementary material for: Modelling the Archipelago: Corfu as a Case Study for a Digital Edition of Cristoforo Buondelmonti’s Liber Insularum  
Source: Open Res Eur. 2024 Jan 8;4:11. [Version 1] doi: 10.12688/openreseurope.16712.1 (PMC11109684; doi:10.12688/openreseurope.16712.1)
Supplement: Peer review history

# Reviewer report: Reviewer response for version 1

**DOI:** 10.21956/openreseurope.18050.r38845

**Published:** 2024-05-13

**Authors:** Anna Foka

**Affiliations:** 1 Uppsala University, Uppsala, Sweden

First of all, thank you for the opportunity to read and review this article. It was a very interesting read and I must admit that I it was for the most part complete, with in terms of the humanities merits of such a case study but also in terms of computation .First of all, thank you for the opportunity to read and review this article. It was a very interesting read.

**A Summary**

****

 This article deals with the digital deliverables of the project Mapping the Aegean: Cristoforo Buondelmonti's Liber Insularum and the Birth of Classical Archaeology"  a three-year research project focused on studying Buondelmonti's 15th-century work Liber Insularum, which is considered the first guide to the Greek islands. The project aims to highlight the book's significance in the rediscovery and exploration of ancient Greece, a region largely unknown to Western European scholars at the time. A key component of the project is creating a digital edition of the text based on a manuscript copy from the Gennadios Library in Athens. This digital edition is envisaged to include an English translation, a detailed geographical, historical, and archaeological commentary, and interactive features for accessing maps and annotations. The digital format allows for representing heterogeneous data from various disciplines and providing an interactive user experience. While a critical edition of the text is desirable, the project's primary goal is not a philological analysis. Instead, it aims to enhance the understanding of the Liber Insularum as a document of early travels to the Greek islands and its role in paving the way for the archaeological rediscovery of ancient Greece. 

**Some major points**:

 1.     The article describes in great detail the very design of the digital edition; it is designed to be an accessible gateway for classical archaeologists, scholars of classical studies, Byzantinists, historians, and others interested in the Greek islands. 

 It is very clearly articulated that the purpose of the digital edition of the Liber Insularum is to create a comprehensive, open, and modular digital resource for studying this important 15th century text by Cristoforo Buondelmonti.

 2.     The article discusses in great detail well the key goals of the digital edition project. 

 -       To recover and preserve the legacy material of the Liber Insularum, which is considered the first guide to the Greek islands. 

 -       To remodel the content into a wider and more generic data and software architecture, allowing for growth and community contributions over time. 

 -       To provide standard digital outputs ranging from web applications, API access, TEI document exports, and semantic web data - making the content accessible to both scholars and general audiences. 

 -       To design the digital edition in an abstract and modular way, so that the methodological approach can be applied to other scholarly scenarios beyond just the Liber Insularum. 

 In summary, the digital edition aims to preserve and expand access to this important historical text through innovative digital methods, while also developing a reusable model for creating complex and open digital scholarly resources.

 In its conception and articulation the article is very interesting as an exercise but there is a couple of shortcomings that, while minor need to be addressed. 

**Some minor shortcomings:**

 1.     The article leaves out the importance of Linked Open Data in its conception and implementation most digital projects attempt to do. While discussing linking information, it does not provide adequate descriptions of how the digital edition supports of linked open data. 

 2.     Another shortcoming of this article is the lack of literature review concerning relevant projects. The authors are welcome to peek on the following articles, which by no means make a comprehensive list but treat different issues of the same project. These articles are also not of a philological character and might be proven useful to the author.  

 Barker. E et al;  2023 [Ref 1]

 Foka, A.,et al, 2023 [Ref 2]

 Foka, A et al.  2022[Ref 3]

 Foka, A.et al; 2021[Ref 4] 

 Barker, E et al, 2020.[Ref 5]

 3.     Lastly, there are several repetitions and colloquial phrases such as  several instances of ’of course’. That said, these even more minor shortcomings and with a fresh proofreading they could be mitigated. 

 4.     It would be great if us readers can learn more about the future of this project- if possible!

Is the case presented with sufficient detail to be useful for teaching or other practitioners?

Partly

Is the work clearly and accurately presented and does it cite the current literature?

Partly

If applicable, is the statistical analysis and its interpretation appropriate?

Yes

Are all the source data underlying the results available to ensure full access and reuse by other researchers?

Partly

Are the conclusions drawn adequately supported by the data and analysis?

Yes

Is the background of the case’s history and progression described in sufficient detail?

Yes

Reviewer Expertise:

Digital Humanities, GIS, Cultural Heritage, Digital Editions.

I confirm that I have read this submission and believe that I have an appropriate level of expertise to confirm that it is of an acceptable scientific standard.

## References

[1] : Journeying through Space and Time with Pausanias’s Description of Greece. *https://www.literarygeographies.net/index.php/LitGeogs/article/view/316* .2023;

[2] : A DIGITAL PERIEGESIS:.2023; 10.2307/jj.2430371.13 205-224 10.2307/jj.2430371.13

[3] : Visualizing Pausanias’sDescription of Greece with contemporary GIS. *Digital Scholarship in the Humanities* .2022;37(3) : 10.1093/llc/fqab093 716-724 10.1093/llc/fqab093

[4] : Information and Knowledge Organisation in Digital Humanities.2021; 10.4324/9781003131816 10.4324/9781003131816

[5] : Coding for the Many, Transforming Knowledge for All: Annotating Digital Documents. *PMLA/Publications of the Modern Language Association of America* .2020;135(1) : 10.1632/pmla.2020.135.1.195 195-202 10.1632/pmla.2020.135.1.195

# Reviewer report: Reviewer response for version 1

**DOI:** 10.21956/openreseurope.18050.r38846

**Published:** 2024-05-07

**Authors:** Melina Tamiolaki

**Affiliations:** 1 University of Crete, Rethymno, Crete, Greece

The article under review is the product of research conducted in the framework of a MSCA project, the aim of which is to produce a digital edition of the work of Cristoforo Buelmondi, *Liber Insularum*, written in the 15th century AD. In this paper, Benedetta Bessi and Daniele Fusi (hereafter the authors) present a case study: the application of a digital tool, Cadmus, for the island of Corfu.

 The article is overall well written and well argued. It presents a useful introduction about the life and works of C. Buelmondi, stressing the need for a new edition, since, as the authors note, no critical edition of *Liber Insularum* is at present available. Hence, the digital edition proposed fills an important gap. In particular, Benedetta Bessi has a strong archaeological background and has already worked on this topic in the traditional format. The expansion of her work to the Digital Humanities is most useful and welcome: she chooses to produce a digital edition (rather than a traditional one), because, through the development of digital tools, this edition will reach a larger audience, being interactive, incorporating complex and diverse information (geographical, ethnographical, archaeological, etymological etc.) and enabling complex queries. 

 The article presents two digital tools, Proteus and Cadmus. The authors are to be commended for their remarkable mastery of these tools; in fact, the paper could alternatively be structured according to these tools, which are described with sufficient clarity. The article is also enriched by several Figures. The first tool (p. 6-10), Proteus, can dissect input format into a sequence of entries; interestingly, this tool can be used to extract text from DOCX. The major focus of the article is the second tool, Cadmus, which functions as a content editor, “a sort of hub where both imported and edited, external resources can be leveraged, and virtually any kind of output can be generated” (p. 10). Through Cadmus, users can add layers of information to a digital edition (e.g. on geography, ethnography, etymology, commentary etc.). Cadmus’ data can also be exported to XML and RDF Format. It is important that Cadmus is user friendly and even people who do not possess advanced IT skills can profit from it. The authors declare that “all data underlying the results are available as part of the article and no additional source data are required” (p. 23).

 The authors talk on various instances about the multi-disciplinary character of this project. However, these references are rather incidental. It would perhaps be more useful for readers (especially teachers and practitioners) to explain this interdisciplinarity right at the beginning (i.e. in the introduction). In this interdisciplinary framework, several disciplines are indeed involved, such as history, archaeology, geography, ethnography, computer science, *and philology* (my emphasis). I find the authors’ claim that “philological analysis of the text remains beyond the scope” of the project (p. 5) a bit misleading, since, for instance, etymology and commentary, which are included in the analysis, are philological tasks. In sum, I think that the topic of interdisciplinarity could have been better clarified.

 Overall, this is an excellent article: it lays the ground for a very interesting and useful digital edition of *Liber Insularum*, which could further serve as a model for other digital editions in the future. 

 Some minor points to improve: 

**p. 3: **Further, the intrinsically open-ended nature of any digital edition lends itself to potentially relevant expansions, either from the contribution of single, specialized scholars, **and** from interested or local communities. 

To reformulate as: Further, the intrinsically open-ended nature of any digital edition lends itself to potentially relevant expansions, either from the contribution of single, specialized scholars, **or **from interested or local communities.

**p. 4: **Even though the Gennadios the mountains are rendered in brown and black is used for the outline of the islands and other geographical features (including mountains), the limited color range appears still close to the original trichromy…

 The syntax is a bit unclear in this phrase; do the authors mean “even though **in** the Gennadios the mountains are rendered in brown and black, **which **is used for…”? 

**p. 13: **Of course, the main practical issue here is that Cadmus is has multi-layer architecture. The word “is” should be deleted.

**p. 13, end**: it seems that the description here fits with Figure 4, rather than with Figure 5.

**p. 15, paragraph starting with “For instance”**: I think that Figure 5 could be added here.

**p. 16, Figure 6**: the image would require a better resolution.

**p. 21, Figure 14**: the image would require a better resolution.

Is the case presented with sufficient detail to be useful for teaching or other practitioners?

Yes

Is the work clearly and accurately presented and does it cite the current literature?

Yes

If applicable, is the statistical analysis and its interpretation appropriate?

Not applicable

Are all the source data underlying the results available to ensure full access and reuse by other researchers?

Yes

Are the conclusions drawn adequately supported by the data and analysis?

Yes

Is the background of the case’s history and progression described in sufficient detail?

Yes

Reviewer Expertise:

Classics; Ancient History; Digital Humanities

I confirm that I have read this submission and believe that I have an appropriate level of expertise to confirm that it is of an acceptable scientific standard.

# Reviewer report: Reviewer response for version 1

**DOI:** 10.21956/openreseurope.18050.r39159

**Published:** 2024-04-15

**Authors:** Eric Poehler

**Affiliations:** 1 University of Massachusetts Amherst, Amherst, Massachusetts, USA

The case study article, "Modelling the Archipelago: Corfu as a Case Study for a Digital Edition of Cristoforo Buondelmonti’s *Liber Insularum*" is a well written and well presented discussion of the the CADMUS tool and its application to the specific instance, the exposition of the island of Corfu. The text is clear on its goals, thoughtful in what level of detail it provides (enough to understand the points being made, but not so much as to be lost in meta-discussion), and overall successful in meeting those goals. Indeed, I appreciated that the article did not attempt a technical documentation of CADMUS and instead, on occasion, elevated to higher philosophical issues that drove the application's underlying design. 

 More information and demonstration of how CADMUS helped with processing of data in the middle step - the creation of annotation layers between legacy data import and structured data export - would have been interesting to read. This is especially true since so little of the Liber Insularum was shown (even relatively little of the one island case study) to illustrate the tool. How does the scholar building the historical data and surrounding metadata do their work? What kinds of affordances and constraints exists in CADMUS? I understand, however, that his was not the primary goal of the paper and perhaps was a causality of word count limits. 

 There are only a few very minor issues of language to suggest for consideration: 

 1.) Egina is more commonly spelled Aegina in English language publications. 

 2.) There are some grammatical misapplications of the word "right"  in a several contexts. For example:

      "So, it is right the consideration "

      "It is right this conversion"

      "it is right the genre"

 It seems something closer to 'proper' (from proprio?) is meant in this instances, but English requires more words and/or a different word order to make this expression. It is a credit to the authors that this is the level of criticism required of their contribution.

Is the case presented with sufficient detail to be useful for teaching or other practitioners?

Yes

Is the work clearly and accurately presented and does it cite the current literature?

Yes

If applicable, is the statistical analysis and its interpretation appropriate?

Not applicable

Are all the source data underlying the results available to ensure full access and reuse by other researchers?

Partly

Are the conclusions drawn adequately supported by the data and analysis?

Yes

Is the background of the case’s history and progression described in sufficient detail?

Yes

Reviewer Expertise:

I am a specialist in Roman Archaeology and Digital Humanities.

I confirm that I have read this submission and believe that I have an appropriate level of expertise to confirm that it is of an acceptable scientific standard.
